# 超高效液相色谱-四极杆-飞行时间质谱法快速辨识芪玉三龙汤化学成分

**DOI:** 10.3724/SP.J.1123.2020.10016

**Published:** 2021-07-08

**Authors:** Mengwen HUANG, Huan WU, Wei YU, Ying WANG, Fengcan WANG, Chunchun ZHANG, Longsheng ZHOU, Zegeng LI

**Affiliations:** 1.安徽中医药大学科研实验中心, 安徽 合肥 230038; 1. Scientific Research & Experiment Center, Anhui University of Chinese Medicine, Hefei 230038, China; 2.中药研究与开发安徽省重点实验室, 安徽 合肥 230012; 2. Anhui Province Key Laboratory of Research and Development of Chinese Medicine, Hefei 230012, China; 3.中药复方安徽省重点实验室, 安徽 合肥 230012; 3. Anhui Province Key Laboratory of Chinese Medicinal Formula, Hefei 230012, China; 4.安徽省教育厅中医药防治肺系重大疾病重点实验室, 安徽 合肥 230038; 4. Key Laboratory of Prevention and Treatment of Major Pulmonary Diseases with Traditional Chinese Medicine, Department of Education of Anhui Province, Hefei 230038, China

**Keywords:** 超高效液相色谱-四极杆-飞行时间质谱, 数据非依赖性采集, 靶向筛查, 芪玉三龙汤, ultra high performance liquid chromatography-quadrupole time-of-flight mass spectrometry (UPLC-QTOF-MS), data independent acquisition (DIA), targeted screening strategy, Qi-Yu-San-Long decoction (QYSLD)

## Abstract

利用超高效液相色谱-四极杆-飞行时间质谱(UPLC-QTOF-MS)的数据非依赖性采集(DIA)技术,结合靶向筛查方法,快速辨识芪玉三龙汤(Qi-Yu-San-Long decoction, QYSLD)化学成分。以Waters ACQUITY UPLC BEH C_18_柱(100 mm×2.1 mm, 1.7 μm)为色谱柱,流速为0.2 mL/min,柱温为35 ℃,进样量为2 μL,以0.1%(v/v)甲酸水溶液-乙腈为流动相进行梯度洗脱。采用电喷雾电离(ESI)源,以全信息串联质谱(MS^E^)技术在正、负离子模式下分别采集QYSLD复杂组分的质谱数据。通过检索文献和在线数据库,建立QYSLD中各单味药材化学成分库。将采集到的样品原始质谱数据与QYSLD化学成分库导入天然产物后处理筛查(UNIFI)平台;在UNIFI平台中设置参数(保留时间偏差为±0.1 min,精确质量数偏差阈值为±5×10^-6^,正离子模式下,选择[M+H]^+^和[M+Na]^+^为准分子离子或加合离子,负离子模式下,选择[M-H]^-^和[M+HCOO]^-^)。经UNIFI平台对MS^E^模式下采集的质谱数据与自建数据库中成分作靶向筛查,结合化合物准分子离子、质谱裂解途径及部分对照品进行结构确认。从QYSLD中共识别出166种化学成分,其中皂苷类22种,生物碱类13种,黄酮类27种,萜类32种,氨基酸类20种,苯丙素类16种,有机酸类9种,甾醇类6种,蒽醌类6种,其他类15种。其中16种成分使用对照品作验证。研究建立的方法能够快速、可靠的表征QYSLD中的化学成分,为该复方的药效物质及质量控制研究奠定了基础。

## Abstract (en)

Qi-Yu-San-Long decoction (QYSLD) is a classic traditional Chinese medicine prescription consisting of ten types of herbal medicines, including Astragali Radix, Polygonati Odorati Rhizoma, Scolopendra, Pheretima, *Solanum nigrum* L., *Hedyotis diffusa* Willd., Coicis Semen, *Euphorbia helioscopia* L., Curcumae Rhizoma, and Fritillariae Cirrhosae Bulbus, combined in a ratio of 15:5:3:3:10:10:10:3:5:3 by weight. QYSLD has been used to treat non-small cell lung cancer (NSCLC) for over 20 years in clinical practice, and its curative effect is considered credible. However, the chemical constituents of QYSLD have not been revealed because of their complexity, which has significantly hindered the systematic clarification of the efficacy of the materials and quality evaluation. In this study, a reliable strategy based on the data-independent acquisition (DIA) technology of ultra high performance liquid chromatography-quadrupole time-of-flight mass spectrometry (UPLC-QTOF-MS) combined with a targeted screening method was established to investigate the chemical components of QYSLD. A 2-μL aliquot from each vial was injected into a Waters ACQUITY UPLC BEH C_18_ column (100 mm×2.1 mm, 1.7 μm) to separate complex components. The temperature of the column was 35 ℃, and the flow rate was set at 0.2 mL/min. The mobile phase consisted of 0.1% formic acid aqueous solution and acetonitrile. Detection was conducted using an Xevo G2-XS QTOF-MS with a LockSpray capable-electrospray interface. The data for complex components in QYSLD were collected by full-information tandem mass spectrometry (MS ^E^) in the positive and negative ion modes. In the MS^E^ mode, data acquisition was performed using a mass spectrometer by rapidly switching from a low-collision-energy (CE) scan to a high-CE scan during a single LC run. Thus, accurate precursor and fragment ions were collected in a single run, which was helpful for the structural elucidation of multiple components in QYSLD. In addition, systematic information on isolated chemical compounds was collected and distinguished from the ten individual herbs in QYSLD using databases such as China Academic Journals Full-text database (CNKI), PubMed, Web of Science, Medline, and ChemSpider. Accordingly, a self-building library of QYSLD, including the component name, molecular formula, and structure of the components from the herbs, was established. Subsequently, the raw MS^E^ data of the collected samples and the self-building chemical composition library were imported into a natural product post-processing screening (UNIFI) platform for targeted screening of the chemical components in QYSLD. The parameters for UNIFI platform were as follows: the retention time deviation was ±0.1 min; an error margin of no more than 5×10 ^-6^ for the identified compounds was allowed; positive adducts, including [M+H]^+^and [M+Na]^+^, were selected; and negative adducts, including [M-H]^-^ and [M+HCOO]^-^, were selected. The results showed that a total of 166 compounds were initially identified, including 22 saponins, 13 alkaloids, 27 flavonoids, 32 terpenes, 20 amino acids, 16 phenylpropanoids, 9 organic acids, 6 sterols, 6 anthraquinones, and 15 other components. Among them, sixteen components were confirmed unambiguously with the reference substances. To better understand the chemical contribution of individual herbs to the entire decoction, the attributes of each component were summarized. This study provides a foundation for exploring the pharmacodynamic substances of QYSLD.

芪玉三龙汤(Qi-Yu-San-Long decoction, QYSLD)是由黄芪、玉竹、天龙、地龙、龙葵、白花蛇舌草、薏苡仁、泽漆、莪术、川贝母十味中药组方而成,是临床用于治疗非小细胞肺癌(NSCLC)的名老中医验方^[1]^。现代药理学研究^[2,3,4]^表明,QYSLD抑制NSCLC的作用可能与其调控PI3K/Akt/mTOR和Wnt/*β*-catenin信号转导通路分子表达有关。虽然QYSLD能有效治疗NSCLC,但其发挥抑制NSCLC作用的物质基础尚未明确。中药(复方)中的复杂化学组分具有多靶点和协同效应的治疗作用特点,对这些复杂化学组分进行解析是揭示中药(复方)药效物质基础并对其进行质量控制的关键步骤。因此,有必要建立一种快速、可靠的分析方法全面表征QYSLD中的复杂化学成分。

近年来,具有高灵敏度、高分辨率的超高效液相色谱-四极杆-飞行时间质谱(UPLC-QTOF-MS)已广泛用于中药(复方)化学成分的分离和结构表征中^[5,6]^。质谱数据采集主要分为数据依赖性采集和数据非依赖性采集(DIA),其中DIA技术无需对样品中的化合物作预选择,而是直接采集经色谱柱分离的所有化合物的质谱信息^[7,8]^。作为典型的DIA技术之一,全信息串联质谱(MS^E^)是一种能够实现“低碰撞能”和“高碰撞能”交替扫描以获得高精确的母离子及碎片离子信息,并能够依据母离子和碎片离子的色谱行为进行关联归属的数据采集方法^[9]^。由于中药(复方)众多成分之间含量差异大、结构类型和理化性质多样,传统人工解谱费时耗力;在存在基质背景信号干扰的情况下,质谱响应信号低或含量很少的成分在MS^E^模式中的信号贡献较小,这些成分在分析鉴定过程中,常易产生漏判。因此,依托于天然产物后处理筛查(UNIFI)平台的靶向筛查方法便成了很好的补充。UNIFI平台可极大减轻以往质谱数据分析的工作量,能对MS^E^低能量下采集的数据自动进行离子流提取和分子式确定,并与自建库中成分作靶向筛查,然后根据MS^E^高能量下的碎片信息推导化合物裂解途径^[10,11]^,最后在预设的过滤条件下输出识别成分的结构信息,对中药及复方中的化学成分进行快速识别。

本研究在前人研究的基础上构建了QYSLD化学成分库,并将其导入UNIFI平台;然后利用UPLC-QTOF-MS的DIA技术采集样品信息,再结合靶向筛查方法快速辨识QYSLD的化学物质基础,共辨识出166种化合物,并通过对照品对其中16种主要化学成分予以确认。该研究将为下一步研究QYSLD中发挥抑制NSCLC的药效成分奠定基础,同时也为其他中药(复方)成分分析提供方法参考。

## 1 实验部分

### 1.1 仪器、试剂与材料

Acquity I Class型UPLC-Xevo G2-XS型QTOF-MS,配有UNIFI平台(美国Waters公司), KQ-500DB型数控超声波清洗器(昆山市超声仪器有限公司), RE-3000A型旋转蒸发仪(上海亚荣生化仪器厂), Milli-Q超纯水净化系统(美国Millipore公司)。

QYSLD(安徽中医药大学第一附属医院院内制剂)。对照品:黄芪甲苷(批号:MUST-19091308,纯度99.8%)、黄芪皂苷I(批号:MUST-20042906,纯度99.1%)、黄芪皂苷II(批号:MUST-20051008,纯度99.8%)、毛蕊异黄酮(批号:MUST-19120901,纯度99.8%)、毛蕊异黄酮-7-*O*-*β*-D-葡萄糖苷(批号:MUST-200920,纯度99.8%)、澳洲茄碱(批号:MUST-20042004,纯度99.6%)、澳洲茄边碱(批号:MUST-19102621,纯度99.4%)、对香豆酸(批号:MUST-20050603,纯度99.9%)购于成都曼思特生物科技有限公司,贝母辛(批号:B20082,纯度98%)、亚油酸(批号:B21421,纯度98%)、鸟嘌呤(批号:B20906,纯度98%)、次黄嘌呤(批号:B20211,纯度98%)购于上海源叶生物科技有限公司,芦丁(批号:100080-201409,纯度99.9%)、精氨酸(批号:140685-201707,纯度99.9%)、脯氨酸(批号:140677-201507,纯度99.9%)、硬脂酸(批号:190032-201001,纯度99.9%)购于中国食品药品检定研究院。甲酸(美国Sigma-Aldrich公司),甲醇和乙腈(德国Merck公司)。

### 1.2 混合对照品溶液的制备

精密称取16种对照品适量,用甲醇溶解定容,配制成对照品储备液。取各对照品储备液10 μL,用甲醇稀释定容,配制成各成分质量浓度约为10 μg/mL的混合对照品溶液,过0.22 μm的微孔滤膜。

### 1.3 样品前处理

QYSLD样品溶液的制备:称取黄芪30 g、玉竹10 g、天龙6 g、地龙6 g、龙葵20 g、白花蛇舌草20 g、薏苡仁20 g、泽漆6 g、莪术10 g、川贝母6 g。加入1340 mL水,浸泡1 h,武火煮沸后文火煎1.5 h,滤过;残渣加入1072 mL水,武火煮沸,文火煎40 min,滤过;合并两次煎煮液浓缩至1 g/mL。取2.5 mL浓缩液与7.5 mL 95%乙醇混合,涡旋混匀,避光放置12 h,取上清液减压离心挥干,残渣加甲醇振摇溶解,定容至50 mL,摇匀,用0.22 μm微孔滤膜过滤后装入进样小瓶。

空白溶液的制备:除不加药物外,其他步骤与QYSLD样品溶液相同。

### 1.4 液相条件

色谱柱:Waters ACQUITY UPLC BEH C_18_柱(100 mm×2.1 mm, 1.7 μm),柱温:35 ℃,流动相:A为0.1%(v/v)甲酸水溶液、B为乙腈,流速:0.2 mL/min。梯度洗脱程序:0~7 min, 3%B~15%B; 7~11 min, 15%B; 11~21 min, 15%B~25%B; 21~26 min, 25%B~35%B; 26~36 min, 35%B~55%B; 36~45 min, 55%B~73%B; 45~51 min, 73%B~85%B; 51~56 min, 85%B~95%B; 56~61 min, 95%B; 61~62 min, 95%B~3%B; 62~65 min, 3%B。进样量:2 μL。

### 1.5 质谱条件

离子源为电喷雾电离(ESI)源,分别采用正、负离子模式检测,实时校正液为亮氨酸脑啡肽。离子源温度120 ℃;扫描范围为*m/z* 50~1200,毛细管电压3.0 kV(正离子模式)、2.5 kV(负离子模式);锥孔电压40 kV;脱溶剂温度350 ℃;脱溶剂气体流速:600 L/h。MS^E^低碰撞能量为6 eV, MS^E^高碰撞能量为20~35 eV。

### 1.6 QYSLD化学成分数据库的建立

通过检索CNKI、Medline、PubMed、Chemicalbook和ChemSpider等数据库,收集、整理QYSLD中十味中药所含化学成分的信息(包括351种化学成分名称,分子式及结构式),结构式使用ChemDraw绘制,文件格式保存为mol格式。

### 1.7 数据分析

将MS^E^低碰撞能量(获得准分子离子或加合离子精确质量数)、高碰撞能量(设置碰撞能量区间,获得特征碎片离子、中性丢失等二级质谱信息)所采集到的QYSLD样品溶液、空白溶液质谱信息和1.6节下建立的QYSLD化学成分库均导入UNIFI平台。UNIFI阈值设定:二维检测最小峰面积为200; 三维检测的低、高碰撞能量下峰强度分别设置为500和150;保留时间偏差为±0.1 min;精确质量数偏差为±5×10^-6^;正离子模式下,选择[M+H]^+^和[M+Na]^+^为准分子离子或加合离子;负离子模式下,选择[M-H]^-^和[M+HCOO]^-^。在上述阈值的过滤条件下对待测成分的离子峰进行自动辨识、校正并输出结构信息。然后结合准分子离子、碎片离子的精确质量数及部分对照品对化合物结构进行确认(具体结果见表1)。

**表 1:** 芪玉三龙汤化学成分的质谱数据及鉴定结果

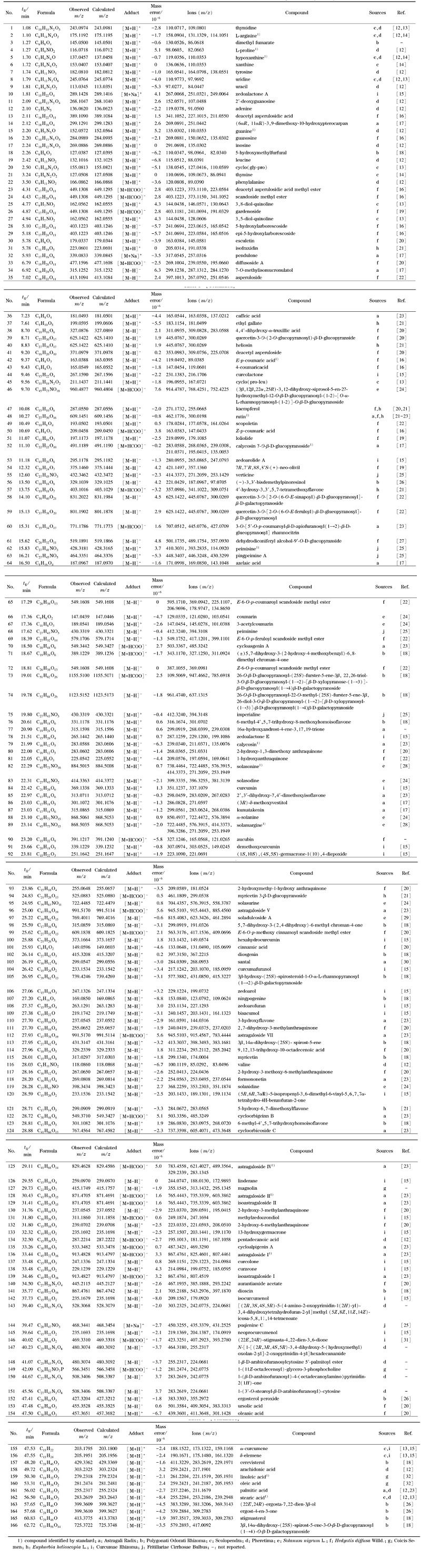

## 2 结果与讨论

### 2.1 QYSLD化学成分分析

利用UPLC-QTOF-MS中的MS^E^模式采集空白溶液、QYSLD样品溶液及混合对照品溶液在正、负离子模式下的质谱信息,得到各自的总离子流色谱图(见图1)。根据1.7节下的数据分析方法对QYSLD中的化学成分进行定性分析,共识别出166种化合物。其中皂苷类22种,生物碱类13种,黄酮类27种,萜类32种,氨基酸类20种,苯丙素类16种,有机酸类9种,甾醇类6种,蒽醌类6种,其他类15种。通过对照品确认了其中16种成分,具体结果见表1。

**图 1:**
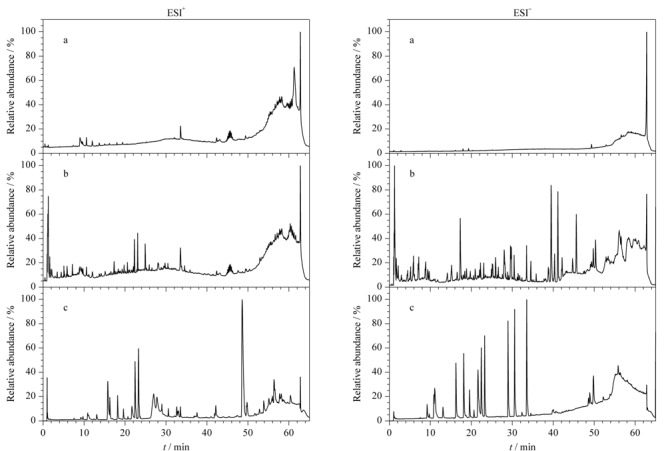
(a)空白溶液、(b)QYSLD样品及(c)16种混合对照品的总离子流色谱图

### 2.2 各类化合物的鉴定与分析

2.2.1 皂苷类

多数皂苷类成分至少含有1个糖链,在高碰撞能量下,其主要的裂解模式为糖基连续性丢失^[33]^。检测到的化合物中有15种甾体皂苷类成分和7种三萜皂苷类成分,主要分布于黄芪和玉竹中。以化合物125(表1中序号,下同)为例进行解析,低碰撞能量下,检测到化合物125(*t*_R_=29.11 min)加合离子*m/z* 829.4628 [M+HCOO]^-^,高碰撞能量下接连损失葡萄糖基(Glc, 162 Da)和木糖基(Xyl, 132 Da),产生*m/z* 621.4027 [M-H-Glc]^-^和*m/z* 489.3564 [M-H-Glc-Xyl]^-^碎片离子,随后C-17支链断裂,再脱去H_2_O(18 Da)产生*m/z* 329.2339 [M-H-Glc-Xyl-C_8_H_16_O_3_]^-^碎片离子,*m/z* 329.2339的碎片离子继续丢失CH_3_(15 Da)、O(16 Da),产生*m/z* 283.1345 [M-H-Glc-Xyl-C_8_H_16_O_3_-C_2_H_6_O]^-^碎片离子。经UNIFI平台靶向筛查,结合对照品验证,确定该化合物为黄芪甲苷,裂解途径如图2所示。其他皂苷类成分以类似方式进行解析。

**图 2:**
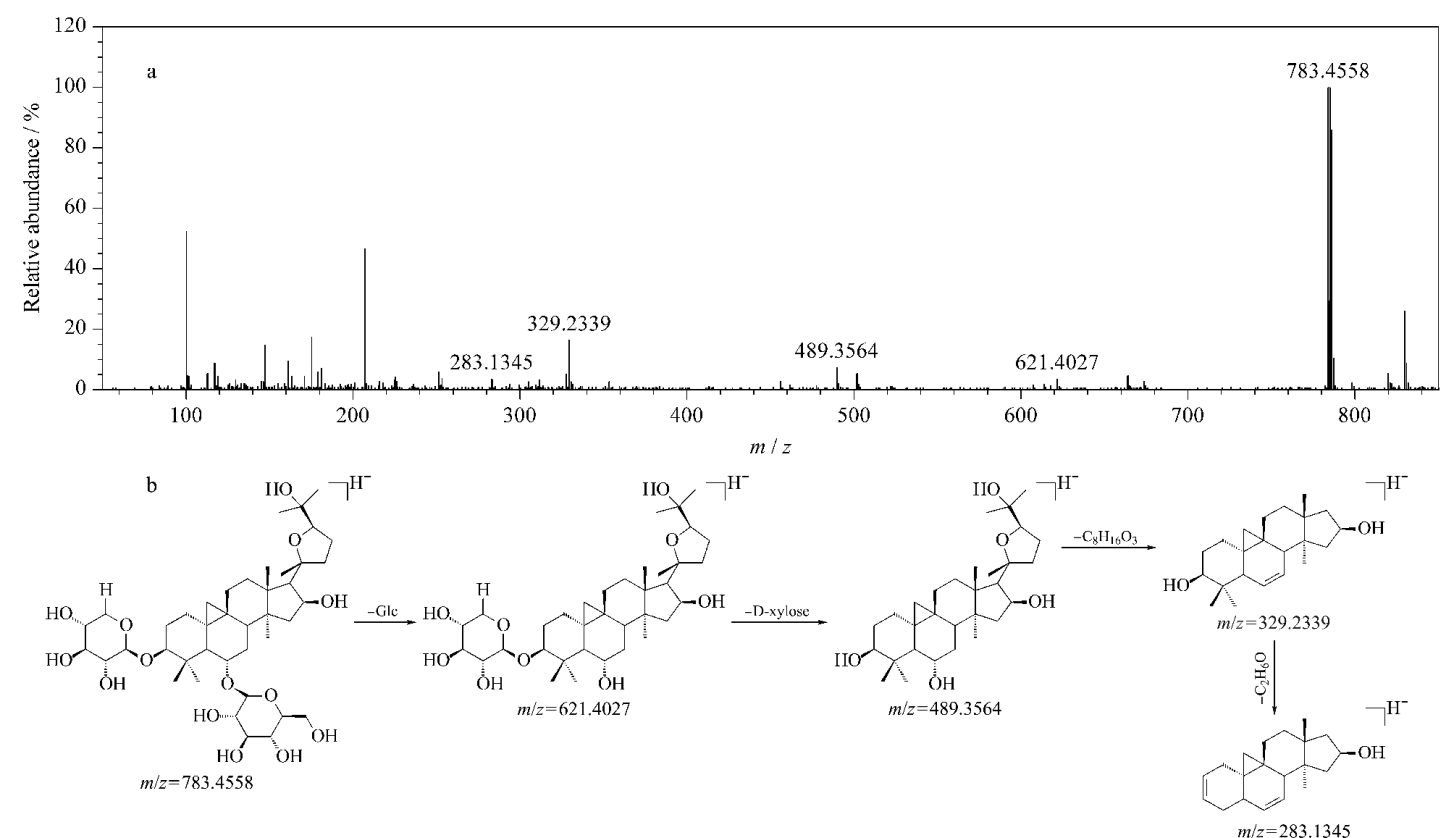
ESI^-^模式下黄芪甲苷的(a)质谱图及(b)裂解途径

2.2.2 生物碱类

生物碱类化合物主要来源于QYSLD中龙葵和川贝母。龙葵中的生物碱大多数为糖苷生物碱。以图1中化合物82(*t*_R_=22.29 min)为例,在正离子模式、低碰撞能量下,检测到准分子离子*m/z* 884.5015 [M+H]^+^,在高碰撞能量下连续脱去鼠李糖基(Rha, 146 Da)、葡萄糖基(Glc, 162 Da)、半乳糖基(Gal, 162 Da),形成*m/z*为738.4464、576.3915和414.3373的碎片离子;通过N规则,可判断*m/z* 414.3373的苷元离子为含1个N原子的生物碱^[34]^,推测该苷元为澳洲茄胺;此外,澳洲茄胺E环断裂产生*m/z* 271.2059和*m/z* 253.1949的特征碎片离子。由此推测化合物82是以澳洲茄胺为苷元,以Rha、Glc、Gal为糖基的澳洲茄碱,这一结果经对照品的*t*_R_和MS^E^数据予以验证,具体裂解信息见图3。

**图 3:**
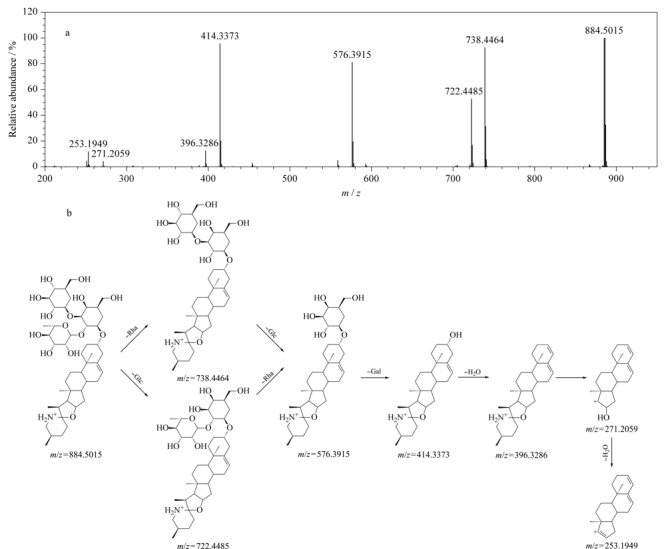
ESI^+^模式下澳洲茄碱的(a)质谱图及(b)裂解途径

其他糖苷生物碱的裂解方式多与澳洲茄碱类似,在正离子模式下产生准分子离子峰[M+H]^+^或[M+Na]^+^,糖链中的糖基连续性丢失形成苷元,苷元环在高碰撞能量下断裂产生特征碎片离子^[34,35]^。根据此裂解规律,结合UNIFI靶向筛查结果,推测化合物88、89和95分别为*α*-茄碱、澳洲茄边碱和澳茄新碱。

川贝母生物碱在正离子模式下有准分子离子[M+H]^+^,在高碰撞能量下常脱去一分子H_2_O(18 Da)产生碎片离子。以化合物62(*t*_R_=15.83 min)为例,[M+H]^+^为*m/z* 428.3181的准分子离子,脱H_2_O产生*m/z* 410.3031的碎片离子,经对照品比对后,鉴定该化合物为贝母辛。另推测化合物55、68和75分别为贝母素甲、贝母素乙和西贝母碱^[36]^。

2.2.3 黄酮类

黄芪、玉竹、白花蛇舌草和泽漆均含有黄酮类化合物。以化合物52为例,在负离子模式下产生加合离子峰[M+HCOO]^-^, *m/z*为491.1189,高碰撞能量下产生*m/z* 283.0588 [M-H-Glc]^-^、*m/z* 268.0365 [M-H-Glc-CH_3_]^-^、*m/z* 239.0308 [M-H-Glc-CH_3_-CHO]^-^、*m/z* 211.0371 [M-H-Glc-CH_3_-CHO-CO]^-^、*m/z* 195.0415 [M-H-Glc-CH_3_-CHO-CO-O]^-^、*m/z* 135.0053 [M-H-Glc-CH_3_-CHO-CO-C_6_H_4_]^-^的碎片离子,推测其为毛蕊异黄酮-7-*O*-*β*-D-葡萄糖苷^[17]^,具体裂解信息见图4。由文献^[37]^可知,黄酮苷在负离子模式下信号较强,在高碰撞能量下易脱去糖基,其苷元进一步丢失H_2_O(18 Da)、CH_3_(15 Da)产生特征碎片离子。依据上述裂解规律,结合UNIFI平台靶向筛查结果,推测化合物48、79、118分别为芦丁、毛蕊异黄酮、芒柄花素^[23]^(具体质谱信息见表1),其中化合物48和79经对照品予以确认。

**图 4:**
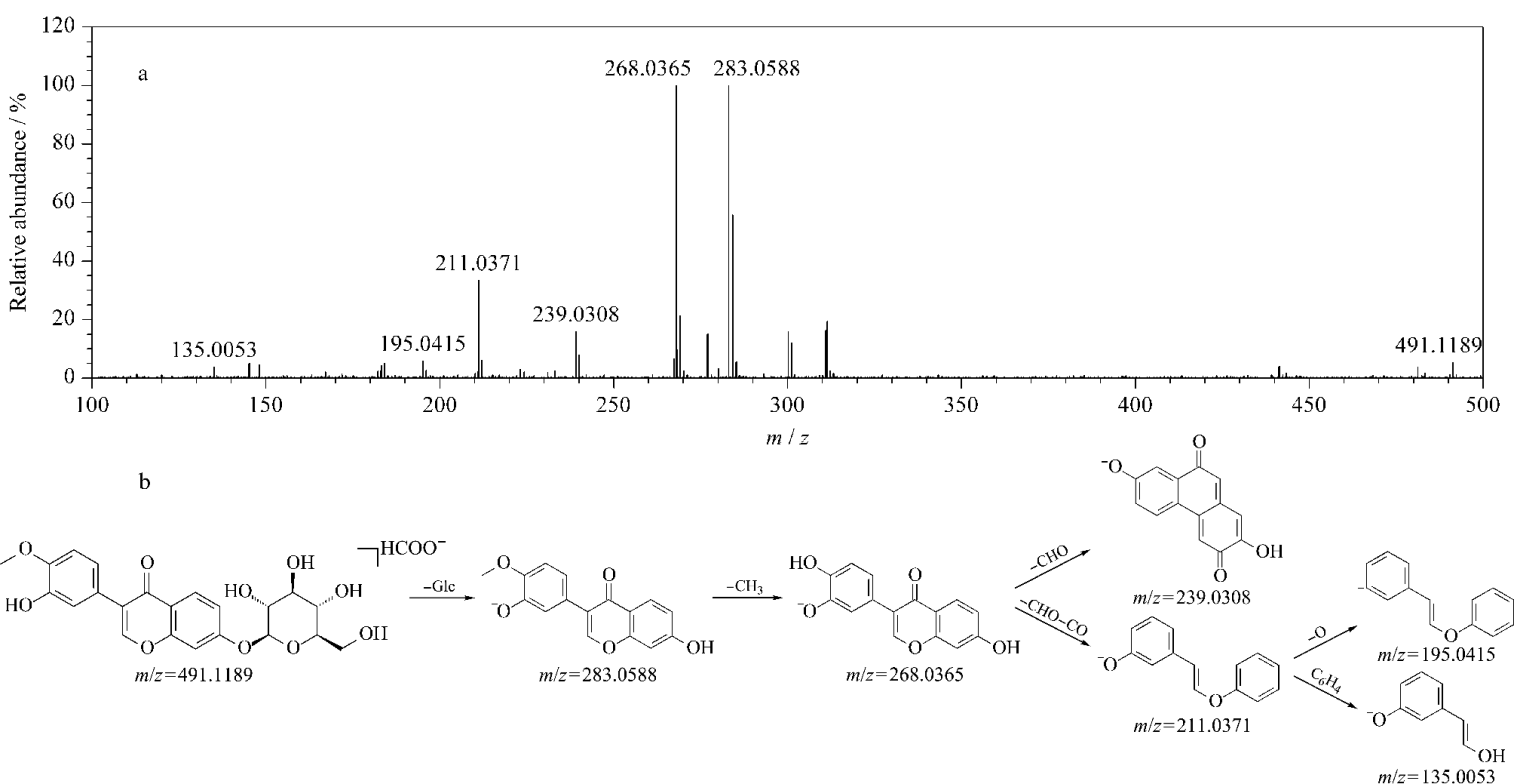
ESI^-^模式下毛蕊异黄酮-7-*O*-*β*-D-葡萄糖苷的(a)质谱图及(b)裂解途径

2.2.4 萜类

萜类化合物共识别出32种,其中17种属于倍半萜类(来源于莪术), 15种属于环烯醚萜类(来源于白花蛇舌草)。环烯醚萜类C-1位常为羟基,且多与糖基结合成苷。环烯醚萜苷类成分裂解所需碰撞能量较小,易产生碎片离子^[22]^,典型特征是吡喃环上易发生Glc(162 Da)的中性丢失,苷元母核结构上常见H_2_O(18 Da)、CO(28 Da)和CO_2_(44 Da)的后续丢失。如化合物65,在低、高碰撞能量下均可观察到准分子离子峰[M-H]^-^, *m/z*为549.1608,以及特征碎片离子*m/z* 369.0942 [M-H-Glc-H_2_O]^-^、*m/z* 225.1107 [M-H-Glc-H_2_O-coumaroyl]^-^、*m/z* 206.9696 [M-H-Glc-H_2_O-coumaroyl-H_2_O]^-^、*m/z* 178.9747 [M-H-Glc-H_2_O-coumaroyl-H_2_O-CO]^-^、*m/z* 134.8650 [M-H-Glc-H_2_O-coumaroyl-H_2_O-CO-CO_2_]^-^,推测该化合物为反式-6-*O*-对香豆酰鸡屎藤苷甲酯。化合物137属于倍半萜类,在低碰撞能量下观察到准分子离子是*m/z* 247.1336 [M+H]^+^,计算分子式为C_15_H_18_O_3_,在高碰撞能量下产生*m/z* 229.1223 [M+H-H_2_O]^+^、*m/z* 214.0984 [M+H-H_2_O-CH_3_]^+^、*m/z* 199.0752 [M+H-H_2_O-2CH_3_]^+^的碎片离子,推测该化合物为姜黄醇酮^[38]^。

2.2.5 苯丙素类

共鉴别出16种苯丙素类化合物。以化合物42为例,其在低碰撞能量下准分子离子为*m/z* 163.0388 [M-H]^-^,在高碰撞能量下脱去CO_2_(44 Da)、C_2_H_6_(30 Da)产生*m/z* 119.0492和*m/z* 89.0385的碎片离子,经对照品确认该化合物为对香豆酸。依据此裂解规律推导化合物36、43、66和101可能是咖啡酸、4-羟基香豆素、香豆素和肉桂酸。

2.2.6 氨基酸类

此类化合物主要存在于天龙和地龙中。由文献^[12]^可知,在正离子模式下,氨基酸类化合物的裂解规律主要是脱去NH_3_(17 Da)和CO_2_(44 Da)。以化合物2(*t*_R_=1.10 min)为例,低碰撞能量下产生准分子离子峰[M+H]^+^, *m/z*为175.1192,通过Masslynx软件推导其化学式为C_6_H_14_N_4_O_2_,高碰撞能量下接连丢失NH_3_和CO_2_,形成特征碎片离子*m/z* 158.0904 [M+H-NH_3_]^+^和*m/z* 114.1051 [M+H-NH_3_-CO_2_]^+^,经对照品确认该化合物为精氨酸。根据裂解方式及对照品的质谱信息指认化合物4、5和15分别为脯氨酸、次黄嘌呤和鸟嘌呤。

2.2.7 有机酸类

有机酸类化合物裂解规律一般是脱去H_2_O(18 Da)、CO_2_(44 Da)和CH_3_(15 Da)等小分子基团。化合物162准分子离子为*m/z* 283.2619 [M-H]^-^,高碰撞能量下产生*m/z* 253.2186 [M-H-2CH_3_]^-^、*m/z* 239.2948 [M-H-CO_2_]^-^的碎片离子,符合上述裂解规律,该化合物为硬脂酸并经对照品验证。依据上述有机酸裂解规律,结合UNIFI平台靶向筛查结果,化合物159、160和161依次推测为亚油酸、油酸和棕榈酸。

2.2.8 其他类

其他类主要是QYSLD中含量种类较少、响应值较小的化合物。通过UNIFI靶向筛查,并结合文献报道^[13,15,18-21]^,共推测出15种化合物(依次为化合物18、25、27、37、38、54、107、120、140、143、147~151),具体质谱信息见表1。

## 3 结论

本研究运用UPLC-QTOF-MS的DIA技术(MS^E^模式)结合靶向筛查方法对QYSLD中复杂的化学成分进行快速、全面的分析,结果从QYSLD中共识别出166个化学成分,其中16个成分经过对照品验证。本研究所建立的方法能够快速、可靠的表征QYSLD中的化学成分,为该复方的药效物质及质量控制研究奠定了基础,同时也为其他中药(复方)所含化学成分的快速解析提供方法参考。但UNIFI平台靶向筛查方法也有不足,如无法区分准分子离子和二级碎片离子均相似的同分异构体。因此,需通过*t*_R_、精确准分子离子质量、碎片离子质量、文献信息及对照品的色谱、质谱信息等对UNIFI平台靶向筛查的可疑化合物进行复核,以排除假阳性结果。
